# Primary Signet Ring Cell/Histiocytoid Carcinoma of the Eyelid: Somatic Mutations in *CDH1* and Other Clinically Actionable Mutations Imply Early Use of Targeted Agents

**DOI:** 10.3390/curroncol28010090

**Published:** 2021-02-16

**Authors:** Lei-Chi Wang, Tai-Chi Lin, Yi-Chen Yeh, Hsiang-Ling Ho, Chieh-Chih Tsai, Teh-Ying Chou

**Affiliations:** 1 Department of Pathology and Laboratory Medicine, Taipei Veterans General Hospital, Taipei 11217, Taiwan; lcwang12@vghtpe.gov.tw (L.-C.W.); ycyeh2@vghtpe.gov.tw (Y.-C.Y.); 2 Institute of Clinical Medicine, National Yang Ming Chiao Tung University, Hsinchu 30010, Taiwan; 3 Department of Ophthalmology, Taipei Veterans General Hospital, Taipei 11217, Taiwan; tclin6@vghtpe.gov.tw (T.-C.L.); cctsai@vghtpe.gov.tw (C.-C.T.); 4 School of Medicine, National Yang Ming Chiao Tung University, Hsinchu 30010, Taiwan; 5 Institute of Biomedical Informatics, National Yang Ming Chiao Tung University, Hsinchu 30010, Taiwan; 6 Department of Biotechnology and Laboratory Science in Medicine, National Yang Ming Chiao Tung University, Hsinchu 30010, Taiwan; 7 Cancer Progression Research Center, National Yang Ming Chiao Tung University, Hsinchu 30010, Taiwan

**Keywords:** signet ring cells, eyelid carcinoma, CDH1 gene, next-generation sequencing

## Abstract

Primary signet ring cell/histiocytoid carcinoma of the eyelid is a rare ocular malignancy and its diagnosis is often delayed. This neoplasm presents as an insidious, diffusely infiltrative mass in the periocular area that later infiltrates the orbit. An exenteration is usually indicated; however, nearly one-third of patients develop local recurrence or metastasis. Morphologically, it resembles signet ring cell carcinoma of the stomach and breast, raising the possibility of mutations in *CDH1*, the gene encoding E-cadherin. To determine whether primary signet ring cell/histiocytoid carcinoma harbors the *CDH1* mutation or other actionable mutations, we analyzed the tumor tissue via next-generation sequencing. We identified only one case of primary signet ring cell carcinoma of the eyelid with adequate DNA quality for sequencing from the pathological archive during the period 2000 to 2020. A comprehensive evaluation including histopathology, immunohistochemistry, and next-generation sequencing assay was performed on tumor tissue. Immunohistochemically, the tumor exhibited E-cadherin membranous staining with the aberrant cytoplasmic staining of β-catenin. Using next-generation sequencing, we demonstrated the mutation in the *CDH1* gene. In addition, other clinically actionable mutations including *ERBB2* and *PIK3CA* were also detected. The alterations in other actionable genes indicate a need for larger studies to evaluate the pathogenesis and potential therapies for primary signet ring cell/histiocytoid carcinoma of the eyelid.

## 1. Introduction

Primary signet ring cell/histiocytoid carcinoma is a rare skin adnexal neoplasm with only fewer than 50 cases reported in the literature [1,2,3,4,5,6,7,8,9,10,11,12,13,14,15,16,17,18,19,20,21,22,23,24,25,26]. While most tumors involve the eyelids, identical neoplasms have rarely been reported in the axilla [27,28,29]. The morphology of the tumor cells resembles carcinoma with a signet ring cell appearance in other solid organs. Previous studies have demonstrated the possible origin of apocrine/eccrine glands with a variable expression of estrogen receptor (ER), progesterone receptor (PR), androgen receptor (AR), and Her2-Neu immunohistochemically [2,3,4,24]. However, the genetic alterations of the tumor had not been determined until a recent publication by Raghavan et al., indicated a mutation in the *CDH1* gene [30] that has been demonstrated in signet ring cell carcinoma of the stomach, breast lobular carcinoma, and plasmacytoid urothelial carcinoma. In this study, we performed a comprehensive mutational investigation using next-generation sequencing to elucidate the mutational profile of the tumor and expand our current understanding of this rare neoplasm.

## 2. Materials and Methods

This study was based on the clinical, histologic, immunohistochemical findings and genetic analysis of a case of primary signet-ring cell carcinoma of the eyelid, which was identified in the archival, institutional files of Taipei Veterans General Hospital. Only two cases were identified during the period 2000 to 2020. The case diagnosed in 2003 was excluded because of the poor quality of DNA for molecular analysis. The study patient was diagnosed in 2019. Clinical information was collected from medical records and ophthalmologists. The patient signed an informed consent for the study which was approved by the institutional review board of Taipei Veterans General Hospital. The resected tumor tissue was fixed in 10% formalin and embedded in paraffin. A panel of immunohistochemical stains including CK7, gross cystic disease fluid protein-15 (GCDFP-15), E-cadherin, Her2-Neu, GATA binding protein 3 (GATA3), AR, ER, and PR were performed for differential diagnosis. The antibodies used, their sources, and their dilutions are given in Table 1. For next-generation sequencing analysis, genomic DNA (gDNA) was extracted from formalin-fixed, paraffin-embedded (FFPE) tissue using the RecoverAll™ Total Nucleic Acid Isolation Kit for FFPE (Invitrogen™, Carlsbad, CA. USA) according to the manufacturer’s instructions. The extracted gDNA was quantified with a Qubit^®^ fluorometer (Life Technologies, Carlsbad, CA. USA) using the Qubit^®^ dsDNA HS Assay Kit; 30 ng of gDNA were subjected to library preparation using the Oncomine™ Tumor Mutation Load Assay (Thermo Fisher Scientific, Waltham, MA. USA), which could simultaneously assess the tumor mutation load and mutation signatures across approximately 1.7 Mb and 409 cancer-related genes. The prepared library was subsequently analyzed using the Ion Library TaqMan^®^ Quantitation Kit (Thermo Fisher Scientific) and the Agilent 2100 Bioanalyzer (Agilent Technologies, Santa Clara, CA. USA) to assess the quantity and quality of the DNA library. A total of 50 pM of a DNA library were subjected to automatic template preparation and chip loading on the Ion Chef System (Thermo Fisher Scientific). DNA sequencing was then performed on the Ion GeneStudio S5 system (Thermo Fisher Scientific). Data were analyzed using the Ion Reporter^TM^ Oncomine Tumor Mutation Load workflow (Thermo Fisher Scientific), and the variants were called when a minimum mean coverage of 500 reads was achieved and at least 5% of the variant reads were identified. The called variants were further filtered using the Taiwan Biobank database to exclude likely benign variants (>1% population allele frequency in the Taiwanese population).

## 3. Results

### 3.1. Clinical Features

**Figure 1:**
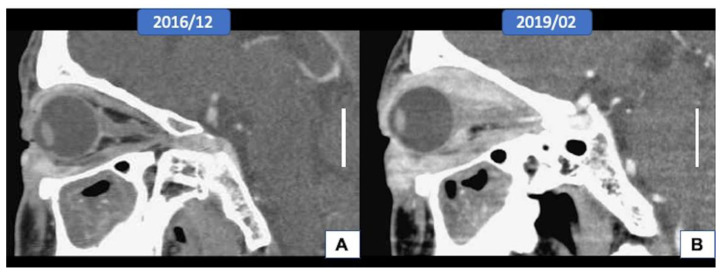
Radiological features of the tumor (scale bar, 2 cm). (**A**,**B**) The orbital computed tomographic scan showed the extension of the tumor from the eyelid to the whole orbital socket after three years.

A 60-year-old man, who had been diagnosed as having poorly differentiated adenocarcinoma of the right eyelid at another hospital for three years, presented with progressive proptosis and limitation of extraocular muscle movement. At examination, the right lower eyelid was diffusely thickened with a nodular consistency. An orbit-computed tomographic (CT) scan showed diffuse enhancing soft tissue infiltrating at the right orbit, involving the right lacrimal gland, the retrobulbar and extraconal space, the eyeball, the extraocular muscle, and the orbital apex, with suspicious extension to the optic canal (Figure 1A,B). A magnetic resonance imaging (MRI) scan of the orbit showed tumor growth in the whole right orbital cavity, causing marked proptosis, encasing the right optic nerve and extraocular muscles, and extending into nasolacrimal duct and outside the orbit to the right cheek. A biopsy of the indurated area of the right lower eyelid revealed infiltration by signet-ring cell carcinoma in the subepithelial stroma. Whole-body PET was performed for a systemic workup and showed tumor growth in the right orbit without other fluorodeoxyglucose (FDG)-avid lesions noted elsewhere in the whole body. Orbital exenteration and craniofacial resection were performed, and a histopathologic study showed extensive orbital extension from the eyelid carcinoma.

### 3.2. Histopathologic Findings

**Figure 2:**
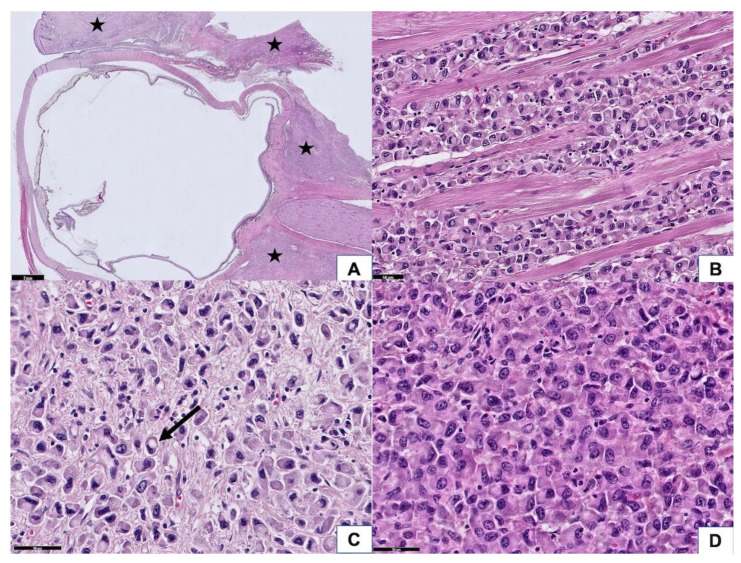
Histopathological features of the tumor. (**A**) At scanning power, the tumor was diffusely infiltrated in the eyelid, extraocular muscle, and retrobulbar soft tissue (asterisk). The optic nerve was spared (50× magnification). (**B**) Tumor cells diffusely infiltrated in the extraocular skeletal muscle, manifesting a destructive growth pattern and causing a limitation of eye movement (200× magnification). (**C**) A few tumor cells reveal a signet ring cell feature (arrow) (400× magnification). (**D**) Some of the tumor cells have enlarged nuclei, exhibiting a histiocytoid appearance (400× magnification).

**Figure 3:**
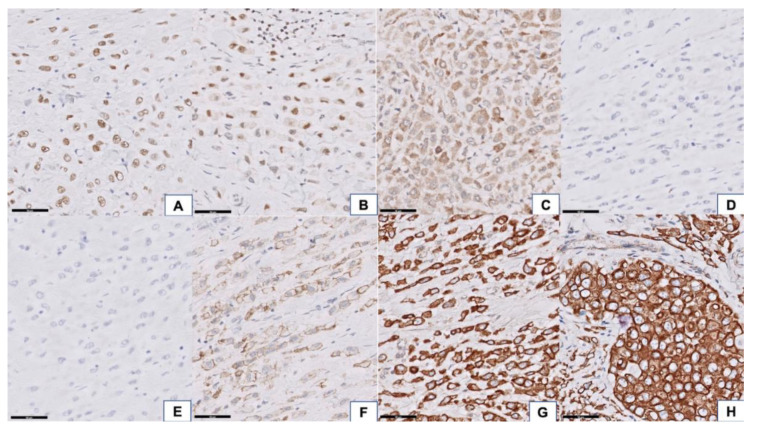
Immunohistochemical features of the tumor. The tumor cells showed focal moderate positivity for androgen receptor (AR) (**A**) 400× magnification, GATA binding protein 3 (GATA3), (**B**) 400× magnification, and gross cystic disease fluid protein-15 (GCDFP15, (**C**) 400× magnification) and negativity for estrogen receptor (ER), (**D**) 400× magnification, and progesterone receptor (PR), (**E**) 400× magnification. Her2-Neu stain exhibited moderate complete staining in 40% of the tumor cells (**F**) 400× magnification. Preserved of E-cadherin membranous staining, (**G**) 400× magnification, with aberrant cytoplasmic expression of beta-catenin, (**H**) 400× magnification, was identified in tumor tissue.

The epidermis of the eyelid was spared, but the full thickness of the dermis was diffusely infiltrated by carcinoma cells. In addition, the tumor extended to the extraocular skeletal muscle, lacrimal gland, sclera, conjunctiva, and part of the cornea. The optic nerve and optic nerve sheath were not involved by the tumor; however, the resection margins in the subcutaneous soft tissue at the cheek area, right orbital apex, and right temporal bone showed tumor cells infiltration (Figure 2A). The neoplastic cells manifested a destructive growth pattern (Figure 2B). Signet ring cell morphology, characterized by abundant eosinophilic cytoplasm and eccentric nuclei, was noted in places (Figure 2C). Some of the neoplastic cells were larger with enlarged nuclei, suggesting a histiocytoid appearance (Figure 2D). Morphologically, this is consistent with the diagnosis of primary signet ring cell/histiocytoid carcinoma of the eyelid. There was no mitotic figures or necrosis identified, which is correlated with its clinically slow-growing behavior. Immunohistochemically, the tumor cells showed a strong-diffuse positivity for CK(AE1/AE3) and CK7 (data not shown). There was moderate-focal reactivity for AR, GATA3, and GCDFP15, whereas no staining was observed with ER and PR (Figure 3A–E). The Her2-Neu stain exhibited moderate complete staining in 40% of the tumor cells on the representative sectioned slide (Figure 3F). The tumor cells showed a preserved of E-cadherin expression. There was aberrant staining for β-catenin being localized in the cytoplasm (Figure 3G,H).

### 3.3. Next-Generation Sequencing

A total of 29 nonsynonymous (missense, splicing site, insertion, deletion) mutations were detected in the tumor tissue of primary signet ring cell/histiocytoid carcinoma of the eyelid, with an allele frequency ranging from 4.41% to 89.43%. In addition to the mutation in *CDH1*, the tumor harbored actionable genetic alterations, including *ERBB2* and *PIK3CA*. The molecular characteristics of the detected mutations are given in Table 2. The nucleotide changes of the *CDH1*, *ERBB2*, and *PIK3CA* genes are shown in Figure S1A–C.

## 4. Discussion

In contrast to the germline mutations in *CDH1* that typify diffuse hereditary gastric cancers [31], our case showed *CDH1* mutation with a variant allele frequency (VAF) of 27.08%, suggesting a somatic mutation in *CDH1*. The splice site mutation of *CDH1* detected in our case has been reported in 27% and 10% of hereditary diffuse gastric cancers and lobular breast cancers, respectively [31], and it constitutes one type of truncating mutations of *CDH1*, which is the most commonly identified mutations in *CDH1*. Compared with the recent publication by Raghavan et al., they also discovered a spice site mutation of *CDH1* (c.531+1 G>T, VAF 23.3%) [30]. Our case represents the second case of *CDH1* mutation reported in the literature.

Although *CDH1* mutations or promotor hypermethylation result in a loss of E-cadherin protein expression in many cancer types, our case revealed the preservation of membranous expression of E-cadherin. This phenomenon has been reported in a proportion of lobular breast carcinoma [32,33,34]. The protein expressed in these cases appears dysfunctional and is not normally associated with the catenin complex. We also demonstrated this by showing an aberrant cytoplasmic expression pattern for β-catenin, suggesting a failed cadherin–catenin complex formation that is required for the maintenance of cell–cell adhesion.

Previous studies based on the genetic analysis of lobular carcinoma of the breast have proposed an evolutionary pathway of neoplastic cells from the E-cadherin-positive to E-cadherin-negative tumor cells. The former harbored less genetic instability and less proliferative index [32]. Another putative pathway involving E-cadherin downregulation is the activation of the TGF-β pathway. Our case exhibited mutations in *CDH1, CDH2* and *CDKN2B*, all of which are genes downstream of the TGF-β pathway, indicating the potential role of the TGF-β superfamily in the tumorigenesis [35]. In summary, *CDH1* alterations and the expression of its encoding protein, E-cadherin, may be modulated by other factors yet to be identified.

Another significant finding is that our case was found to have mutations in targetable kinases, *ERBB2* and *PIK3CA*, similar to the results reported in plasmacytoid urothelial carcinoma [36]. Raghavan et al. also described the presence of targetable mutations in *NTRK3*, *CDKN1B*, and *PIK3CA* in their case of primary signet ring cell carcinoma of the eyelid [30]. Alterations in *ERBB2(HER2)* have been reported in diverse cancers, including breast and gastric cancer. HER2-targeted agents, such as trastuzumab, have been FDA-approved and led to dramatic improvements in outcomes across different malignancies [37]. Our patient carried the somatic *HER2* mutation (p.V777L), which has been reported in breast cancer. This mutation results in activating mutation that is sensitive to the irreversible kinase inhibitor, neratinib [38], which has been approved by the FDA for patients with early-stage HER2-positive breast cancer [39]. The *PIK3CA* gene encodes the phophatidylinosital-4,5-bisphosphate 3-kinase catalytic subunit alpha [40], a catalytic unit of the PI3-kinase (PI3K) pathway. Recurrent somatic mutations in PIK3CA are frequent in cancer and result in the activation of the PI3K/AKT/MTOR pathway. Mutations in PIK3CA are common in many cancer types and are observed in 20–30% of breast, cervical, and uterine cancers and 10–20% of bladder, gastric, head and neck, and colorectal cancers [41,42]. The FDA-approved PI3K inhibitor, alpelisib, has been used for the treatment of patients with breast cancer under certain conditions. A histopathological resemblance between cutaneous apocrine carcinoma and breast carcinoma is well known. Given the presumed apocrine gland origin of primary signet ring cell/histiocytoid carcinoma and the lack of standard guidelines in adjuvant therapy, one might consider treating it based on the general guidelines for the treatment of breast cancer [29]. Previously, anti-estrogen therapy has been used as an adjuvant therapy, with some success in patients of primary signet ring cell/histiocytoid carcinoma with ER expression [2]. Recently, one patient received anti-androgen therapy because of AR expression and the co-existence of prostate cancer. The anti-androgen treatment slowed the effect of primary signet ring cell/histiocytoid carcinoma and reached a “stable disease” for two years [24]. Whether these mutations or hormonal status have true therapeutic relevance in primary signet ring cell/histiocytoid carcinoma requires further investigation.

Our study has only examined one case, however, one must bear in mind that these neoplasms are very rare and are infrequently encountered as histological specimens. Although mutational analysis via next-generation sequencing has been reported in one other case, our case doubles the knowledge of the tumor genetics and supports *CDH1* in the pathogenesis. With more cases reported, a later meta-analysis can be performed to evaluate the impact of these genetic alterations and look for generalizable determinants.

## 5. Conclusions

Primary signet ring cell/histiocytoid carcinoma of the eyelid is a rare entity that is unfamiliar to general pathologists and is often diagnosed as “poorly-differentiated adenocarcinoma, suggesting metastatic adenocarcinoma”. A thorough systemic workup fails to show metastatic disease, indicating a primary malignancy in the orbital area. The morphology of the signet ring cell is reminiscent of signet ring cell carcinoma of the stomach, signet ring cell variant breast lobular carcinoma, and plasmacytoid urothelial carcinoma, suggesting a potential common genetic alteration in the *CDH1* gene. Furthermore, the presence of other clinically actionable alterations in genes such as *ERBB2* and *PIK3CA* and unresectable, local aggressive disease imply that future research should investigate the early use of targeted therapies as a potential treatment for patients with primary signet ring cell/histiocytoid carcinoma of the eyelid.

## Figures and Tables

**Table 1:** Antibodies used in the immunohistochemical study and their results.

Antibody	Clone	Source	Dilution	Case
E-cadherin	HECD1	Invitrogen	1:300	+++
β-catenin	D10A8	Cell Signaling	1:50	+++ (membrane and cytoplasm)
Estrogen receptor	6F11	Leica	1:100	-
Progesterone receptor	16	Leica	1:200	-
Her2-Neu	A0485	DAKO	1:900	++
Cytokeratin	AE1/AE3	Leica	1:200	+++
Cytokeratin 7	RN7	Leica	1:100	+++
GCDFP15	23A3	Novocastra	1:150	++
GATA3	L50-823	ZETA	1:250	++
AR	SP107	ZETA	1:100	++

*Note*: Staining results of the case. Strong positivity (+++), moderate positivity (++), and negative staining(-)

**Table 2:** Mutational signatures of the patient with primary signet ring cell/histiocytoid carcinoma of the eyelid.

Gene	Amino Acid Change	Coding	Locus	Variant Effect	Allele Frequency
TPR	p.(D1348H)	c.4042G>C	chr1:186308883	missense	16.82%
LRP1B	p.(D41N)	c.121G>A	chr2:142567932	missense	4.41%
NFE2L2	p.(F246S)	c.737T>C	chr2:178096594	missense	53.28%
LTF	p.(R23dup)	c.68_69insAAG	chr3:46501284	Inframe insertion	99.95%
PIK3CA	p.(K111_L113del)	c.333_341delGATCCTCAA	chr3:178916943	Inframe deletion	24.73%
TET2	p.(H863Y)	c.2587C>T	chr4:106157686	missense	13.31%
WRN	p.(P982S)	c.2944C>T	chr8:30989999	missense	45.58%
CDKN2B	p.(A38T)	c.112G>A	chr9:22008841	missense	4.00%
TAF1L	p.(M155I)	c.465G>A	chr9:32635113	missense	15.25%
RALGDS	p.(T883I)	c.2648C>T	chr9:135974068	missense	8.05%
SUFU	p.(P18S)	c.52C>T	chr10:104263961	missense	5.56%
EP400	p.(P35S)	c.103C>T	chr12:132445267	missense	4.97%
ERCC5	p.(D703G)	c.2108A>G	chr13:103518170	missense	46.35%
CDH1	unknown	c.687+1G>T	chr16:68842752	splice donor variant	27.08%
FANCA	p.(R591Q)	c.1772G>A	chr16:89845355	missense	89.43%
NLRP1	p.(V1241L)	c.3721G>C	chr17:5424906	missense	55.09%
NLRP1	p.(M1119V)	c.3355A>G	chr17:5433966	missense	51.70%
NLRP1	p.(T995I)	c.2984C>T	chr17:5437285	missense	48.10%
NLRP1	p.(T878M)	c.2633C>T	chr17:5445243	missense	47.70%
NLRP1	p.(T782S)	c.2345C>G	chr17:5461671	missense	52.24%
NLRP1	p.(T246S)	c.737C>G	chr17:5463279	missense	47.73%
ERBB2	p.(V777L)	c.2329G>T	chr17:37881000	missense	13.26%
RNF213	p.(A5021V)	c.15062C>T	chr17:78363034	missense	49.92%
CDH2	p.(E38K)	c.112G>A	chr18:25727697	missense	7.50%
STK11	p.(H168Y)	c.502C>T	chr19:1220409	missense	4.72%
JAK3	p.(G936V)	c.2807G>T	chr19:17942208	missense	9.55%
AXL	p.(I252V)	c.754A>G	chr19:41737174	missense	51.61%
TAF1	p.(V803I)	c.2407G>A	chrX:70607231	missense	7.46%
TAF1	p.(G804S)	c.2410G>A	chrX:70607234	missense	5.97%

## Supplementary Materials

The following are available online at https://www.mdpi.com/1718-7729/28/1/90/s1, Figure S1: Images of CDH1, ERBB2, and PIK3CA gene at specific loci were taken using Integrative genomics viewer (IGV) software.
