# Supplementary material for: Primary Signet Ring Cell/Histiocytoid Carcinoma of the Eyelid: Somatic Mutations in CDH1 and Other Clinically Actionable Mutations Imply Early Use of Targeted Agents
Source: Curr Oncol. 2021 Feb 16;28(1):918–27. doi: 10.3390/curroncol28010090 (PMC7985776; doi:10.3390/curroncol28010090)
Supplement: Supplementary file 1 [file curroncol-28-00090-s001.pdf]

cancers

MDPI

**A** CDH1 c.687+1G>T

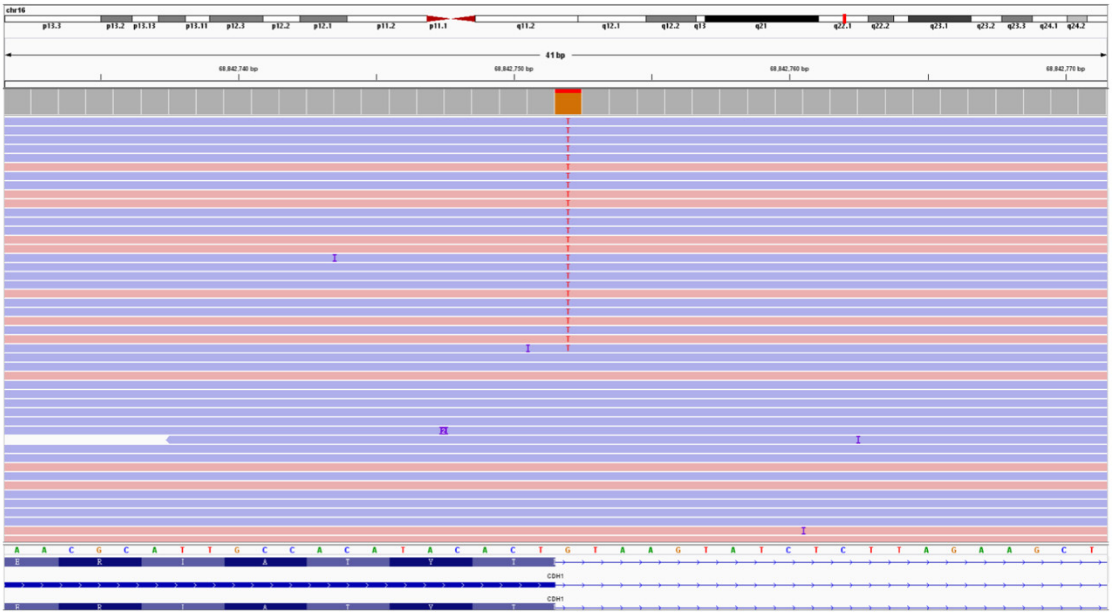

**B** ERBB2 c.2329G>T

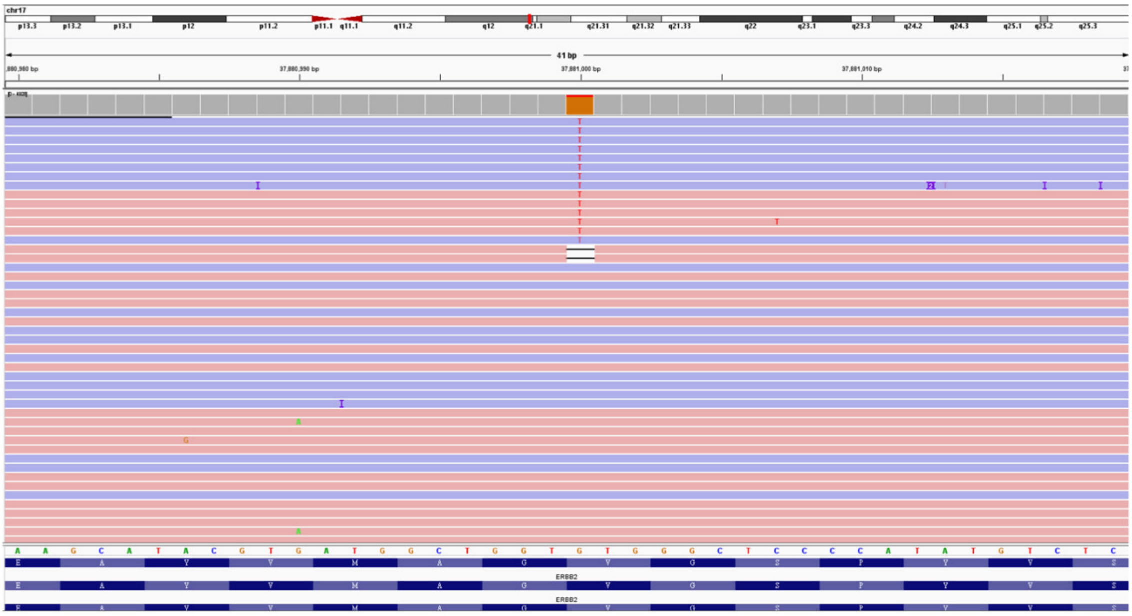

**C** PIK3CA c.333_341delGATCCTCAA

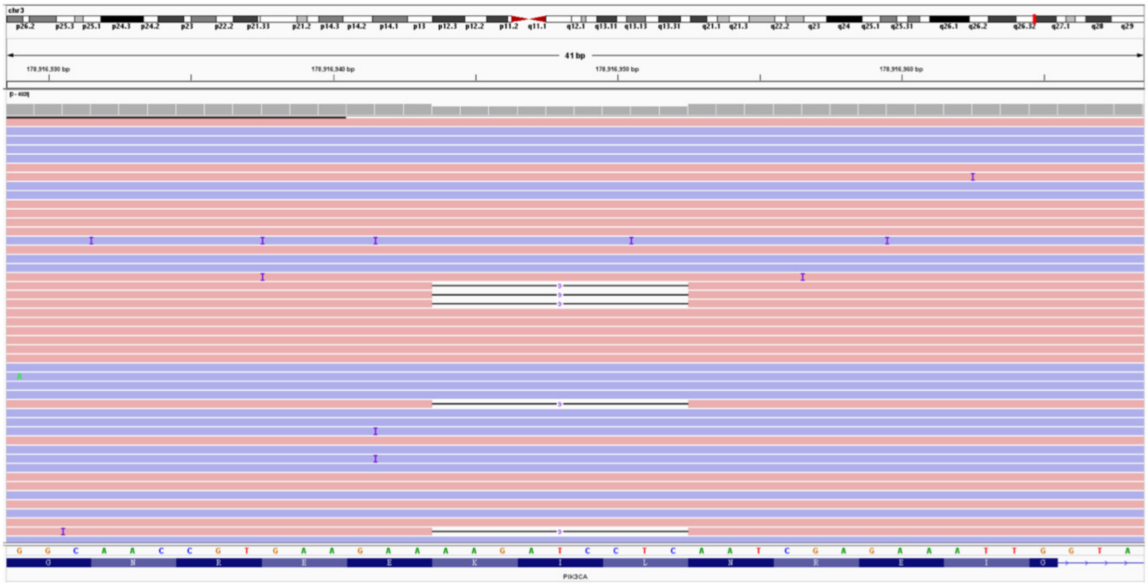

**Supplementary Figure 1.** Images of *CDH1*, *ERBB2*, and *PIK3CA* gene at specific loci were taken using Integrative genomics viewer (IGV) software. (A) In *CDH1*, the sequence revealed a nucleotide change of c.687+1G>T in CDH1 gene, leading to a splice site mutation. (B) In *ERBB2*, the sequence revealed the nucleotide change of c.2329G>T, resulting in a valine-to-leucine substitution at codon 777 in ERBB2. This mutation has been reported in breast cancer as an activating mutation that is sensitive to the irreversible kinase inhibitor, neratinib. (C) In *PIK3CA*, the sequence showed a 9 bp deletion between nucleotides 333 and 341, resulting in an in-frame deletion of PIK3CA protein (p.K111_L113del).
